# Experimental fortification of intestinal anastomoses with nanofibrous materials in a large animal model

**DOI:** 10.1038/s41598-020-58113-4

**Published:** 2020-01-24

**Authors:** Jachym Rosendorf, Jana Horakova, Marketa Klicova, Richard Palek, Lenka Cervenkova, Tomas Kural, Petr Hosek, Tomas Kriz, Vaclav Tegl, Vladimira Moulisova, Zbynek Tonar, Vladislav Treska, David Lukas, Vaclav Liska

**Affiliations:** 1 Department of Surgery, Faculty of Medicine in Pilsen, Charles University, Prague, Czech Republic; 2 Biomedical Center, Faculty of Medicine in Pilsen, Charles University, Prague, Czech Republic; 3 Department of Surgery, University Hospital Regensburg, Regensburg, Germany; 4 Department of Nonwovens, Faculty of Textile Engineering, Technical University in Liberec, Liberec, Czech Republic; 5 Department of Histology and Embryology, Faculty of Medicine in Pilsen, Charles University, Prague, Czech Republic; 6 Department of Anesthesiology and Intensive Care Medicine, Faculty of Medicine in Plzen, Pilsen, Czech Republic

**Keywords:** Implants, Experimental models of disease

## Abstract

Anastomotic leakage is a severe complication in gastrointestinal surgery. It is often a reason for reoperation together with intestinal passage blockage due to formation of peritoneal adhesions. Different materials as local prevention of these complications have been studied, none of which are nowadays routinely used in clinical practice. Nanofabrics created proved to promote healing with their structure similar to extracellular matrix. We decided to study their impact on anastomotic healing and formation of peritoneal adhesions. We performed an experiment on 24 piglets. We constructed 3 hand sutured end-to-end anastomoses on the small intestine of each pig. We covered the anastomoses with a sheet of polycaprolactone nanomaterial in the first experimental group, with a sheet of copolymer of polylactic acid with polycaprolactone in the second one and no fortifying material was used in the Control group. The animals were sacrificed after 3 weeks of observation. Clinical, biochemical and macroscopic signs of anastomotic leakage or intestinal obstruction were monitored, the quality of the scar tissue was assessed histologically, and a newly developed scoring system was employed to evaluate the presence of adhesions. The material is easy to manipulate with. There was no mortality or major morbidity in our groups. No statistical difference was found inbetween the groups in the matter of level of peritoneal adhesions or the quality of the anastomoses. We created a new adhesion scoring system. The material appears to be safe however needs to be studied further to prove itsʹ positive effects.

## Introduction

Anastomotic leakage remains one of the major problems in gastrointestinal (GI) surgery^1,2^. It occurs in 3–19% of colorectal surgeries depending on many factors some of which can and some of which cannot be influenced^3–6^. For rectal anastomoses, the reported risk factors include, besides others, male gender, preoperative radiotherapy and so-called low anastomosis^7^, i.e. an anastomosis within 5 cm from the anus. One of the most important factors influencing the anastomotic healing has proven to be the oxygenation on the site of the anastomosis. Correct oxygen levels are needed for leukocytes activation, fibroblasts production and angiogenesis, which are essential for wound healing^8^. The technique of construction of GI anastomoses is also very important. Although it has been modified and improved repeatedly throughout the history of modern surgery, the mentioned anastomotic leakage as well as other kinds of complications have not been eradicated completely so far (though their frequency has been lowered noticeably). Various sealing techniques and materials were tested both experimentally and clinically including fibrin patches, fibrin glue, collagen patches, hyaluronic acid derivatives and many others^9–16^ as a local barrier protection, while other experiments included systemic administration of various substances^17,18^. The anastomotic leakage occurs in different severity, prolonging the patientsʹ stay in the hospital. Minimal leakage can stay clinically invisible, being detectable only by laboratory markers^19^. The incomplete dehiscence can be approached conservatively by administering antibiotics and parenteral nutrition until the anastomosis is sealed^20–23^. It is usually associated with abdominal drain secretion, abdominal discomfort, temperature elevation, elevation of C-reactive protein and other inflammatory markers^24^. The worst cases need to be approached more radically with relaparotomy bringing a high risk of stomy and higher morbidity and mortality^25,26^.

Peritoneal adhesions (PAs) are bonds between peritoneal surfaces of different abdominal organs. They can range from thin films to thick fibrous bonds containing vessels^27^. Some amount of PAs appears after almost every intraabdominal surgical procedure, but also after inflammations, radiotherapy or chemotherapy^28,29^. PAs can be a source of other complications as they cause abdominal discomfort in many patients, decreasing their quality of life in the long term follow-up. Even more serious problem is abdominal passage blockage, which can appear due to PAs in any time of the patients’ life from the surgery onwards^29,30^. There are various products on the market designed to decrease the level of postoperative adhesions, none of which is commonly used nowadays in GI surgery to successfully prevent the formation of adhesions while maintaining the GI anastomoses healing process intact. Specific inflammatory responses are crucial for both tissue healing after surgical interventions and adhesion formation; so it is therefore a very difficult task to influence the anastomotic healing positively while trying to decrease the amount of newly formed adhesions. To influence one part of the process in one way while the other one in the opposite way seems to be easier by using some kind of barrier protection rather than a systemic treatment^31^.

There are several systems of PA evaluation that focus both on quantity and quality of the adhesions. Coccolini’s peritoneal adhesion index divides the abdominal cavity into segments and evaluates the amount of adhesions in each of them separately, while the other systems evaluate adhesions based on their mechanical properties^32–34^; nevertheless, none of these systems grade regional adhesions in the site of anastomosis in the GI tract.

According to the literature, nanomaterials have never been used in attempts to resolve these complications, therefore we decided to test their ability to reinforce GI anastomoses and investigate their effect on the formation of PAs. In our study we focused on non-woven nanofibrous scaffolds. These structures are considered as very interesting in recent years in the field of tissue engineering thanks to their similar morphology to extracellular matrix^17,18^. Srouji S *et al*.^35^ have noticed their ability to accelerate tissue regeneration in the site of surgery.

There are several ways how to produce nanofibers. The most appealing method for mass production seems to be electrospinning, which is a method with highly adjustable settings allowing to create a large variety of different products with different shapes and thicknesses of the fibers. It is also a method suitable for high volume production^36^. In the process of electrospinning a liquid polymer solution is dosed to an electrode (spinneret) charged with electric potential, while a collector either grounded or oppositely charged is placed on the opposite side of the apparatus. In the presence of electric field, the drop of the polymer shapes into a so called Taylorʹs cone from which the material is dragged to the collector. The stream of the solution gets unstable in the process and the solvent evaporates before the polymer hits the collector. The polymer solution forms different types of structures depending on individual settings. The needle-less spinning has been developed lately, allowing production in larger volumes^36,37^.

Nanomaterials have been experimentally used in numerous surgical applications to fortify various anatomical structures^38–40^. However, the impact of the nanomaterials on healing in the abdominal cavity and on the formation of PAs has not been described yet. The right material for fabrication must be chosen according to the parameters needed. Biodegradability is a crucial quality. Biodegradable polymers are widely used in human medicine; they have been successfully used even as nanofibrous cloths having the best results when the speed of degradation is similar to the speed of tissue regeneration^41^. The wound healing of an anastomosis on the GI tract takes about 3 weeks, from this point on the scar tissue only matures and remodelation of connective tissue continues for much longer^42^. Polycaprolactone and polylactic acid are approved biocompatible polymers with good mechanical properties and cytocompatibility^43^ that are also used for production of different surgical sutures.

Our aim was to investigate whether it is possible to use the nanomaterials in abdominal surgery to determine their influence of the nanomaterials on the healing process of GI anastomosis, and on the formation of PAs. We also aimed to develop a new scoring system for PAs specific for the site of surgery.

## Materials and Methods

### Nanomaterial preparation

Nanomaterials for our experiment were fabricated via electrospinning on the Nanospider^TM^ machine which is a construct of Technical University of Liberec, Faculty of Textile Enigineering, Department of Nonwovens and Nanofibrous Materials, Czech Republic. Electrospinning has been selected as a method for fabrication of planar fibrous scaffolds as this technique allows large scale production of nanofibers; our team has also wide experience with this technique^39^.

We used two types of biocompatible polymers: polycaprolactone (PCL), and a polylactic acid-polycaprolactone copolymer (PLCL). PCL (mean weight = 43000 g/mol, Polysciences) and PLCL (Purasorb PLC 7015, Corbion) were dissolved in chloroform, acetic acid and ethanol solution (8:1:1 volume fractions) to the final concentration of 16% and 10% respectively (concentrations allowing optimal electrospinning properties based on previous research^41^). The solutions were electrospun onto a spun bond (nonwoven cloth underlay) for easy manipulation and application. The material has been sterilised using ethylene oxide (37 °C, Anprolene). This method has been tested before to prove its safety and frugality to the nanomaterial^41^.

### Material characterization

Scanning electron microscopy (SEM) was employed to obtain images of the fibers; the pictures were analyzed as described in previous work of Horakova *et al*.^41^. Materials were also tested *in vitro* for degradability and mechanical properties^41^.

### Experimental design

All experimental procedures with the use of piglets were described in an experimental protocol approved by the Commission of Work with Experimental Animals at the Medical Faculty of Pilsen, Charles University, and were under control of the Ministry of Education, Youth and Sports of the Czech Republic (project code: MSMT-26570/2017-2). All procedures were performed in compliance to the law of the Czech Republic, which is compatible with the legislation of the European Union.

Healthy male and female Prestice black-pied pigs were randomly allocated to 3 groups using simple randomization (8 animals per each group): PCL group, PLCL group and a control group with no material applied. Each animal was given a unique code. All animals were 12–14 weeks old weighing between 19–35 kg.

Prior to the surgery, the animals were weighed, and intramuscularly premedicated with 10 mg/kg of ketamine (Narkamon, Spofa, Czech Republic), 5 mg/kg of azaperone (Stresnil, Jannssen Phramaceutica, Belgium) and 0,5 mg atropine (Atropin Biotika, Hoechst Biotika, Slovak Republic); general anesthesia was then induced and maintained by intravenous administration of propofol (1% mixture 5–10 mg/kg/h Propofol, Fresenius Kabi, Norway). Fentanyl 1–2 μg/kg/h (Fentanyl Torrex, Chiesi cz, Czech Republic) was used for continuous analgesia. Augmentin 1.2 g as an antibiotic prophylaxis was administered intravenously (GlaxoSmithKline Slovakia, Slovak Republic). A ProPort Plastic Venous Access System with PolyFlow polyurethane catheter (Deltec, Smiths medical, U.S.A.) was implanted and introduced through one of the jugular veins.

**Figure 1:**
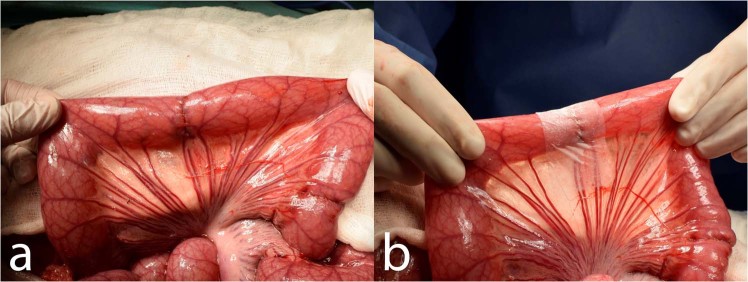
Reinforcing the end-to-end anastomosis on the small intestine in a pig model: (**a**) constructed anastomosis; (**b**) the PCL nanomaterial applied to the site of anastomosis partially covering the mesentery.

We entered the abdominal cavity via an upper middle laparotomy. Three end-to-end anastomoses were constructed on the small intestine in 70, 90 and 110 cm aborally from the duodeno-jejunal junction. We transsected the intestine using monopolar coagulation and constructed a hand sutured anastomosis using MONOSYN 4/0 (Glycolide 72%, Caprolactone 14%, Trimethylencarbonate 14%) double needled polycaprone suture line (B-Braun, Germany), following a standard technique of extramucosal running suture (Fig. 1a). No intestinal resection was performed. A 2 × 5 cm large piece of PCL or PLCL nanomaterial (respecting the group) was placed in the area of the suture, covering the whole surface of the anastomosis (Fig. 1b). No fortifying material was used in the Control group. The intestine was then carefully reposed into the abdominal cavity. Wet swabs were used throughout the procedure for manipulation with the viscera.

The animals were monitored for three weeks by trained blinded caretakers. A fixed realimentation process was scheduled and the ability of the animals to feed according to the schedule was observed. Vomiting was considered as intolerance of the current food dosage. The activity of animals was also monitored.

Blood samples were taken during the experiment at five time points: on day 0 before the application of the material (preoperative baseline sample), exactly two hours after the application of the nanomaterial, on day 7, on day 14, on day 21. Basic biochemical parameters were tracked in these samples (bilirubin, GGT, ALT, AST, ALP, albumin, urea, and creatinine) to see deviations in the animals’ metabolism.

We weighed the animals at the end of the observation period, performed laparotomy in full anesthesia again. We inspected the abdominal cavity for changes, PAs (listed organs involved in the adhesions), checked for the presence of free GI content (signs of anastomotic leakage), intestinal strictures, and intestinal diameter growth (signs of GI passage blockage). We acquired photodocumentation, collected samples of the intestine with anastomoses and fixed them into a 10% buffered formalin (cut in the mesenterial line and pinned onto a cork underlay). The second surgery as well as the macroscopic assessment and sample collection were performed by a blinded surgeon. We sacrificed the animals after the sample collection.

### Scoring of adhesions

**Figure 2:**
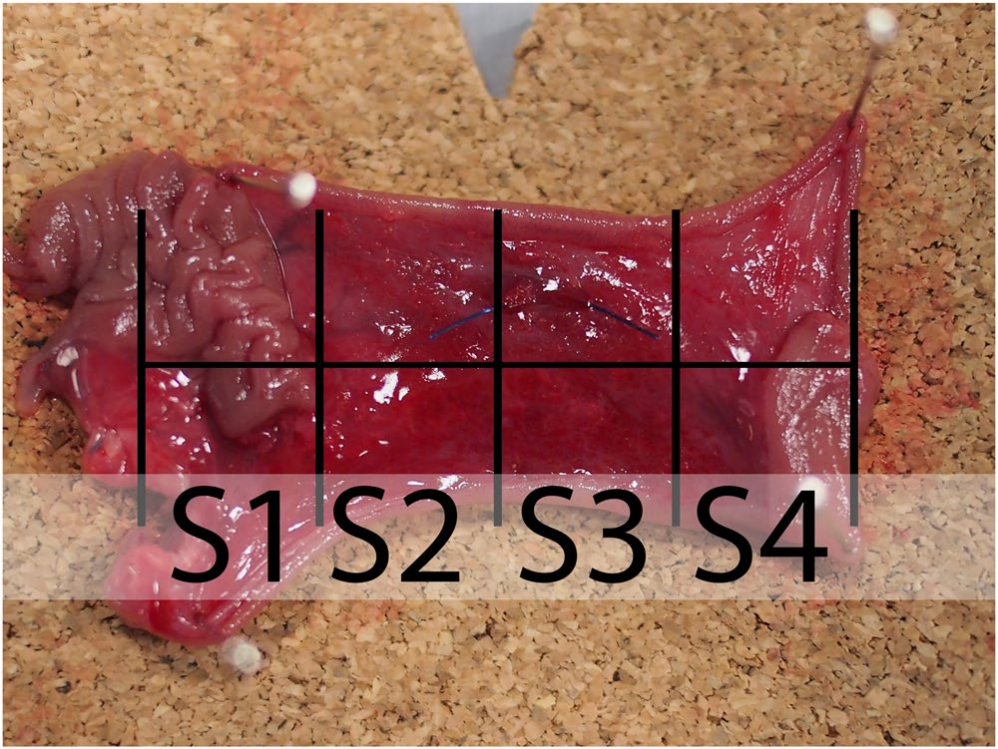
Perianastomotic adhesions amount scoring system for the presence of peritoneal adhesions on the intestine with an anastomosis: The sample (specimen from the Control group) was divided into 4 segments; oral part of the intestine is in the upper side of the image while the aboral part is in the lower part of the image. The area of the suture (the anastomosis itself) is located underneath the horizontal line; scoring of this sample: S1) segment no. 1 scoring 2 points; S2) segment no. 2 scoring 0 points; S3) segment no. 3 scoring 0 points; S4) segment no. 4 scoring 1 point.

None of the quantitative systems of evaluation of PAs were useful for our experiment as we performed surgery only on a small part of the abdominal cavity and the systems usually score the whole abdomen. Thus we created a new quantitative scoring system for our purposes - a *Perianastomotic adhesions amount score* (PAAS). To evaluate the amount of PAs, we divided the area of the specimen into four equal quarters along the circumference of the intestine. Each segment was assigned zero to two points based on the level of adhesions: zero for no adhesions in the segment, one point for adhesions covering the segment partially and two points for adhesions in the whole length of the segment. This resulted in zero to eight points per anastomosis and zero to twenty four points for one animal (in 12 evaluated segments per animal). The specimen were collected carefully together with the surrounding tissue (depending on the level of adhesions), about 4 cm of the intestine was used for each one. The quality of adhesions was evaluated according to the Zühlke’s classification^33^. The intestine was then *ex vivo* transected longitudinally on the mesenteric side, and pinned to a piece of cork. The polarity (oral and aboral part) of the intestine was respected in all measurements (Fig. 2). Each sample was given random alphanumeric code for blinding during the histologic assessment.

### Histology

After fixing, we processed the samples by standard paraffin technique. We stained 4 µm thick sections by hematoxylin and eosin for comprehensive overview; Verhoeffʹs hematoxylin and green trichrome technique was used for staining connective tissues and picrosirius red for visualization of collagen in polarized light. We used immunohistochemical methods for detection of vascular endothelium using Polyclonal Rabbit Anti-Human von Willebrad Factor (A 0082, Dako – Agilent, dilution 1:1000); smooth muscles were detected by Monoclonal Mouse Anti-Human Smooth Muscle Actin (Clone 1A4, M0851, Dako – Agilent, dilution 1:500); for detection of granulocytes and tissue macrophages we used S100A9 Monoclonal Antibody (MAC387, MA1-80446, ThermoFisher Scientific, dilution 1:200).

### Stereology

Microscopic images of IHC samples were stereologically assessed. We defined the reference space as the region of intestinal wall without mucosa located 3 mm proximally and 3 mm distally from the center of the anastomosis (contact of muscle layers); the region includes a suture line. We investigated samples qualitatively and quantitatively.

Volume fractions of endothelial cells, of MAC387 positive cells and of collagen within the reference space were assessed by computerized software system (Stereologer, Stereology Resource Center). The microscope used was Nikon Eclipse Ti-U with, camera Promicra camera.

### Statistical analysis

Standard frequency tables and descriptive statistics were used to characterize the sample data set. Adhesion scores were analyzed with respect to the group, quadrant and anastomosis position in the intestine (1st, 2nd, and 3rd) using repeated measures ANOVA, thus respecting the order and dependency of the three anastomoses sewn in each piglet. The same method was used to assess the differences between groups in collagen and vWF volume fractions. Volume fraction of MAC 387 was considerably affected by random presence of a stitch in some of the slides. Each piglet was therefore assigned one value for anastomoses with stitches (either the mean of all stitch-positive anastomoses, value of a single stitch-positive anastomosis or a missing value if no stitch-positive anastomoses were observed for that piglet) and one value for anastomoses with no stitches (defined analogically). Two-way main-effect ANOVA was then used to evaluate the differences in MAC827 in relation to group and stitch presence. All reported p values are two-tailed and the level of statistical significance was set at α = 0.05. Statistical processing and testing were performed using STATISTICA data analysis software system (Version 12; StatSoft, Inc, 2013; www.statsoft.com).

## Results

**Figure 3:**
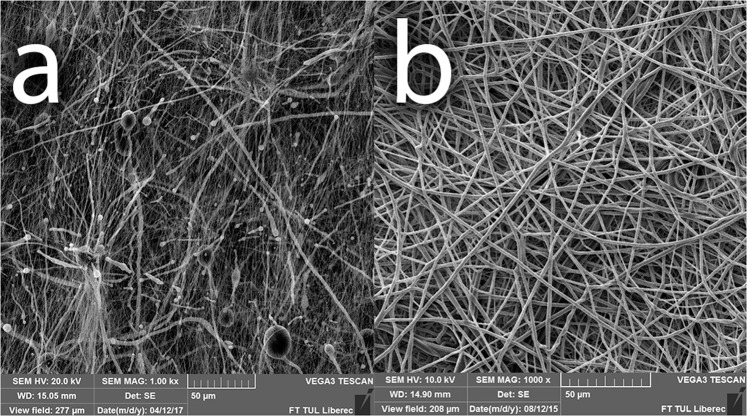
Scanning electron microscopy image of (**a**) the PCL nanomaterial at 1000x magnification and (**b**) the PLCL nanomaterial at 1000x magnification.

Two types of biodegradable nanomaterials for anastomosis fortification testing were prepared by electrospinning, the PCL based sheets (Fig. 3a) and the PLCL sheets (Fig. 3b) (Table 1). The material was electrospun on the spun bond underlay that facilitated easy manipulation while using this material during the surgical procedure.

**Table 1:** Summary of the most important results for each group.

	Control Group (n = 8)	PCL Group (n = 8)	PLCL Group (n = 8)	*p-*value between groups (test)
Material fibre thickness	—	325 ± 36 nm	2047 ± 585 nm	—
Material thickness	—	49 ± 5 nm	53 ± 6 nm	—
Macroscopic signs of anastomotic stenosis (count; %)	0; 0%	0; 0%	0; 0%	—
Macroscopic signs of anastomotic leakage (count; %)	0; 0%	0; 0%	0; 0%	—
Mean PAAS score per segment (0–2) (mean ± SEM across pigs)*	0.479 ± 0.086	0.823 ± 0.171	0.688 ± 0.070	0.715 (repeated measures ANOVA)
Incomplete re-epithelisation (count; %)	0; 0%	0; 0%	0; 0%	—
Volume fraction of vWF positive cells [%] (mean ± SEM)	2.22 ± 0.10	2.16 ± 0.16	2.38 ± 0.12	0.690 (repeated measures ANOVA)
Volume fraction of collagen fibres [%] (mean ± SEM)	15.51 ± 2.10	15.67 ± 2.36	11.87 ± 1.91	0.740 (repeated measures ANOVA)
Volume fraction of MAC387 positive cells [%]:				0.550 (two-way ANOVA)
• stitch not in sample (n: mean ± SEM)	8: 0.38 ± 0.09	8: 0.46 ± 0.19	8: 0.21 ± 0.06	
• stitch in sample (n: mean ± SEM)	7: 0.80 ± 0.23	5: 0.67 ± 0.16	6: 0.70 ± 0.27	

*Each animal was assigned a score equal to the average of all segment scores in that animal (i.e. 24 segment scores per animal; result theoretically ranging from 0 to 2). Mean and Standard error of the mean (SEM) stated in the table were then calculated from these animal averages.

We successfully created a model of intestinal anastomosis on pig with use of PCL and PLCL nanofibrous scaffolds.

Both types of material are easy to peel of the spun bond underlay, they can be then easily manipulated with. They adhere to the intestine and hold well on the intestinal wall, yet they can also be rearranged when needed. No further fixation of the materials was necessary.

All animals survived the whole length of the experiment. None of the animals developed either ileus or sepsis. Two animals from the PCL group vomited single time, so the realimentation schedule was not respected in their case. It is worthy to note though they tolerated the feeding from then on. All animals from the Control group and the PLCL group were able to feed according to the schedule, with daily stool and no signs of gastrointestinal passage blockage. The activity of the animals was not decreased in any of the groups throughout the postoperative observation. We did not notice any case of infection of intravenous port in the PCL group; one case of infection of the port appeared in the PLCL group, and two cases were in the Control group. We also noticed one laparotomy wound infection in one of the animals in the PLCL group in a form of an abscess. No intervention was needed.

There were no significant differences in the observed biochemical parameters between the groups and no remarkable deviations from healthy animals (baseline blood sample).

All animals managed to maintain their weight within the range of 5% of their preoperative weight.

**Figure 4:**
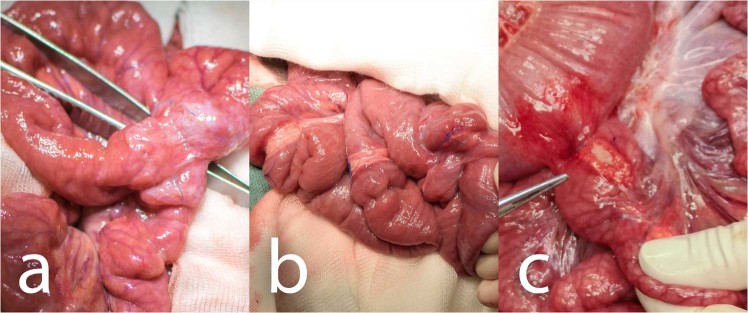
Macroscopic findings in animals of different groups: (**a**) Control group, two anastomoses adhered together, oral parts are marked with a blue suture; (**b**) PCL group, all three anastomoses are visible, the material is clearly visible in the site of application; (**c**) PLCL group, pointing at one of the anastomoses adhering to the colon, oral part is marked with a blue stitch.

There were no signs of GI content leakage within the second surgery in any of the animals (free GI content, thick peritoneal fluid, fibrin films) (Table 1). All of the anastomoses could be found in the reoperation, the nanomaterial remained fixed, covering the suture line completely in all of them. It has been neither absorbed nor dislocated. All anastomoses were sufficient, no visible defects were found in any of them (Fig. 4a–c). PAs involving other organs than the small intestine were found in 3 animals from the Control group, in 5 animals from the PCL group and in 5 animals from the PLCL group. Most of these were adhesions of the left median liver lobe to the incision scar.

**Figure 5:**
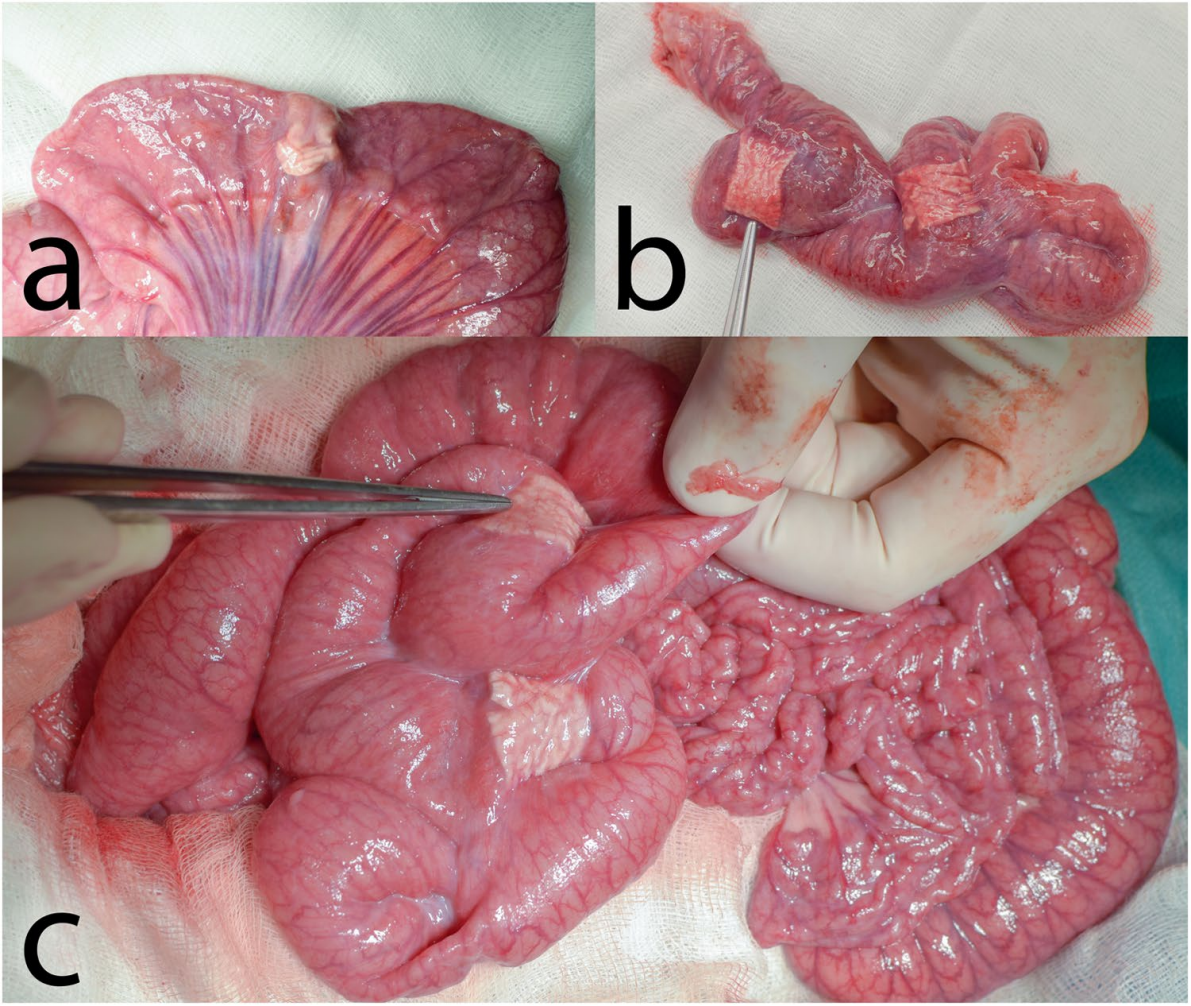
Anastomoses after 3 weeks: (**a**) typical appearance of the small intestine on the 21st postoperative day (PCL group); most of the intestine seems intact with no adhesions, the segments involved in anastomoses are more or less in adhesions, the diameter of the intestine is larger in the proximal segments of the intestine; the material is clearly visible and not dislocated; (**b**) severe adhesions in another animal from the PCL group; (**c**) adhesion free intestine in a different animal from the PCL group.

**Figure 6:**
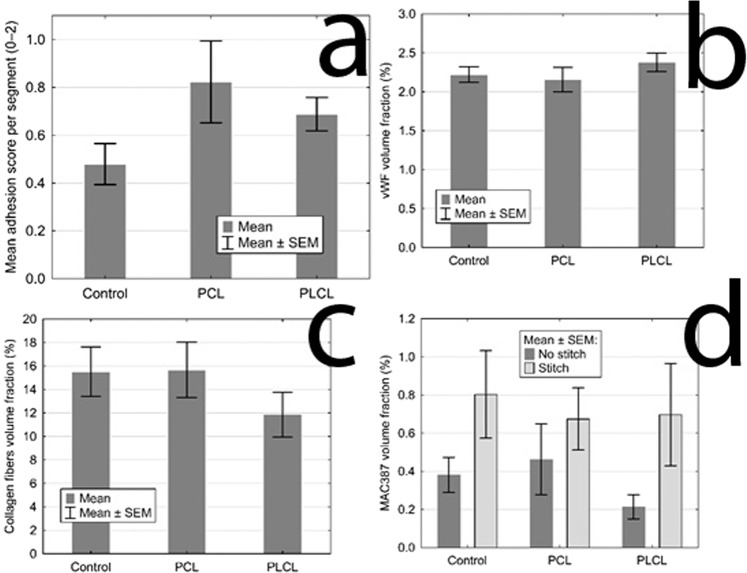
Statistical analysis of quantitative assessment of different parameters: **(a**) The mean adhesion score for each group, the control group scored the lowest with no statistical significance; (**b**) The volume fractions of vWF positively stained area for each groups showing the level of vascularisation, the three groups show the same quality of scar in this aspect; (**c**) The volume fractions of collagen fibres for each group, the three groups show the same quality of scar in this aspect; (**d**) The volume fractions of MAC387 positive area for each group, showing the inflammatory cells infiltration, the presence of a stitch in the section proves to be the only statistically significant factor, the three groups show the same quality of scar in this aspect as well.

The severity of PAs was largely variable within the groups. There was some amount of adhesions in almost all animals in the site of the surgery. Typically, the most adhesions were located within the area of the intestine we manipulated with, connecting the small intestine to the surrounding tissues, mostly only with the small intestine itself (Fig. 5a–c). The adhesions were relatively evenly spread within this area, not surrounding the intestinal circumference in the area of our material or suture line predominantly. The organs we did not manipulate with were usually adhesion-free. The perianastomotic adhesions were in all animals grade Zühlke 2 if present. The adhesions had to be separated by sharp dissection; no clear vascularization was macroscopically visible, though. Only one animal (from the PCL group) didn’t develop any perianastomotic adhesions, this was recognized as grade Zühlke 0. The least adhesions according to our scoring system were found in the Control group, ranging from 2 points to 11 per animal (46 points for 8 animals in total), then 66 points for the group PLCL in total (4–11 points per animal) and 79 points for group PCL (0–16 points per animal). Statistical analysis showed these differences between groups as non-significant (p = 0.715) (Fig. 6a) (Table 1). The position of the anastomosis (first, second or third) also proved not to be a significant factor (p = 0.490) for the amount of adhesions. The most important parameter showed to be the segment of anastomosis while the inner segments (2 and 3) did not show a lot of adhesions, the segments 1 and 4 tended to be heavily adhered (p˂0.001).

Almost all animals exhibited some level of dilatation of the proximal segments of the small intestine; we observed it in all 8 animals in the PLCL group, in 7 animals in the PCL group, and also in 7 animals in the Control group. Nevertheless, the difference between the groups was not statistically significant.

**Figure 7:**
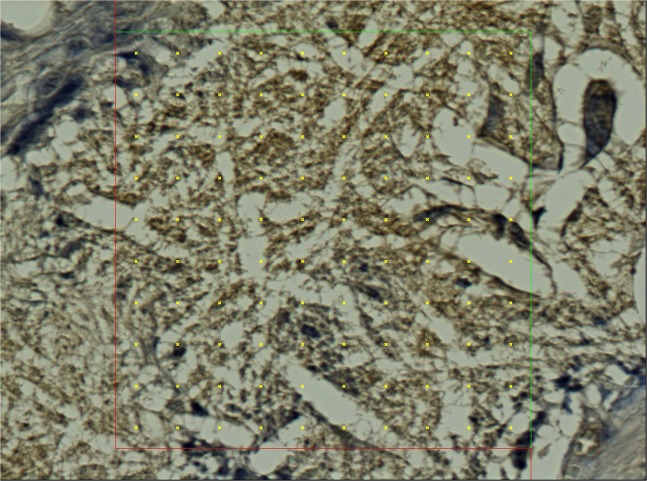
Histological section, PCL group, MAC387 staining: Detail of the marginal zone of the material applied, the empty spaces in the shape of the fibres (stereological grid).

**Figure 8:**
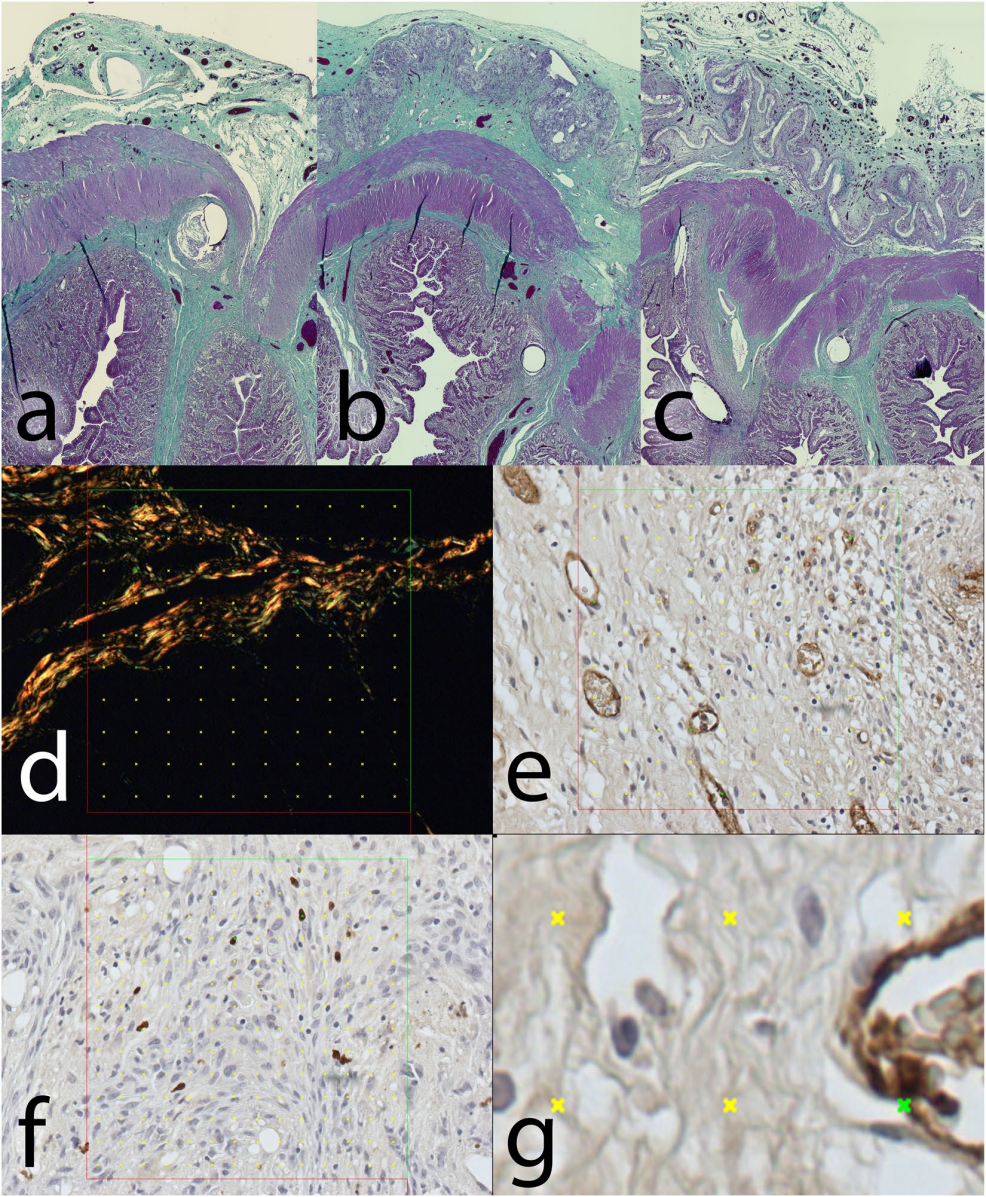
Histological staining of explanted anastomoses: **(a**) Green trichrome: Control group; (**b**) Green trichrome: PCL group, the empty space on the site of application of the nanomaterial can be seen in the upper layer, surrounded by normal granulation tissue; **(c**) Green trichrome: PLCL group, a much thinner empty area can be seen in the upper layer, also surrounded by normal granulation tissue; (**d**) PSR staining, collagen fibres stained yellow, stereological mesh; (**e**) vWF factor staining, the endothelial cells stained brown, stereological grid; (**f**) MAC 387 staining stereology, positive cells stained blue, stereological grid; (**g**) magnification of vWF staining stereology with a positive cross in the upper right corner.

The material was washed out of the sections during the histological staining process. The presence of the material could be detected on the sections as an empty space surrounded by granulation tissue with a borderline of tissue permeated with empty spaces in the form of single fibers (Fig. 7). We observed no morphological abnormalities in standard histological stainings (Fig. 8), all physiological layers were present in all samples. Also the successful reepithelisation was found in all samples (Table 1). The volume fraction of von Willebrand factor positive cells (endothelial cells) did not show statistically significant differences between the groups (p = 0.690) (Fig. 6b), nor did the volume fractions of collagen (p = 0.740) (Fig. 6c) and neutrophiles with macrophages (p = 0.550) (Fig. 6d) (Table 1).

The section areas containing suture material exhibited significantly higher level of inflammatory cells infiltration than the stitch free sections (p = 0.001) (Fig. 6e).

## Discussion

To the best of our knowledge, we were the first to use the PCL and PLCL nanofibrous scaffolds in this kind of application. We successfully designed a study to determine whether the material can be used for this purpose. There have been experimental works focusing on utilization of different locoregional types of protection in the site of intestinal anastomosis^9–16^, yet no use of nanofibrous scaffolds has been described so far.

We found the material to be very easy to handle and to apply onto the intestine. The fact that there is no need of further fixation to the viscera is very positive as the application form can be a limiting factor when it comes to translation into the clinical practice. The fibrin glue could be an example of material that unnecessarily prolongs the surgery time as it needs to dry for 10 minutes before the surgeon can reinsert the viscera into the abdominal cavity^14,44^.

We had no mortality in our study and also no major complications. There were also no clinical changes observed that would suggest development of sepsis or ileus, and the animals managed to maintain their weight; therefore we assume the material does not contribute to postoperative GI obstruction. Most patients develop an anastomotic leakage within the first 2 weeks after the surgery^45,46^, we covered three postoperative weeks of observation.

Nordentoft *et al*. experimented with fibrin coated collagen patches (TachoSil, fibrin sealant) in a pig model of intestinal anastomosis. Two anastomoses per animal were performed on the small intestine after a resection of 2 cm of the intestine. There were no significant differences between the experimental and the Control group in this study in terms of morbidity, mortality and sings of anastomotic leakage as well^10^. In the second study the usage of the same material on a colonic anastomosis showed notable reduction of anastomotic leakage^47^. However, when it comes to clinical use of this material, the fibrin glue does not seem to promote healing as the same authors stated in their review article including 28 studies, only 7 of which revealed a positive effect of the glue^48^. Recently, a clinical study was designed to determine the effect of TachoSil patch in human patients^12^. The study subjected the patients after resection surgery for colorectal cancer to application of the patch over the constructed anastomosis on the large intestine, but was terminated after each of the first eight patients met with complications of different severity. The study concluded that the microbiome of the anastomosis is altered negatively by covering the anastomosis with any kind of material, but did not support this hypothesis with any data.

According to macroscopic findings we can describe the anastomoses from our experiment as well healed. Moreover, we did not observe any morphological changes either in the surrounding tissues or in the whole abdominal cavity, and thus the material seems to be safe to use. The question of the degradation speed remains unanswered at this moment as the material used was always present in the place of application at the end of the experiment. This suggests the material does not have a tendency to slip away but it is also not absorbed as fast as expected. For example a complete reabsorption of the fibrin glue has been described after variable periods ranging from 7 to 20 days^14^.

We used the very new *Perianastomotic adhesions amount score* we developed, which accurately describes the quantity of adhesions in the site of an anastomosis on a circular hollow viscus. Some of the systems used in clinical practice consider also the quality of the adhesions, however they evaluate the whole abdominal cavity^32,33^. We did not aim to evaluate the quality macroscopically and mechanically as we assessed the tissue histologically.

The differences in the amount of PAs between the groups were not significant, which can be due to a small size of our experimental groups. The absolute numbers suggest the material increases the amount of PAs in itsʹ surroundings. This can be caused by easy infiltration of the material by peritoneal fibroblasts from both sides of the material because of its structure similar to extracellular matrix as Srouji described earlier^35^. There was also certain amount of adhesions involving different organs than the small intestine, but there was no clinical manifestation associated with those that we know about.

The amount of postoperative adhesions in the peritoneal cavity has been successfully decreased both experimentally and clinically using different substances, usually in a form of gel. Hyaluronic acid based gels or also polycaprolactone based gels can serve as examples^31,49–51^; however, the influence of such materials on the anastomotic healing has not been described and therefore cannot be considered safe in our application.

A very important factor for the formation of adhesions is the material the viscera are manipulated with. Dry swabs damage the peritoneum, cause inflammation and, consequently, adhesion formation^52^. We used wet swabs throughout our experiment. The formation of adhesions should be finished by the end of the three week observation period (although their characteristic may change during the time after this period and the problems they cause can appear much later^42^. To evaluate the clinical impact of all the adhesions formed a longer observation period would be needed.

Even though the materials were dissolved during the process of histological staining, the area of application was clearly visible under a microscope as a tissue-free layer. We followed a standard system of assessment of the healing of an intestinal anastomosis described in different studies^12^. No morphological or any other statistically significant differences were found between the groups using this system. We value this result as positive since we assume that the healing process was not affected in a negative way and that the resulting scar is of the same quality as a physiologically healed one. The system does not evaluate peritoneal adhesions formation, though. As the peritoneal adhesions are certainly a source of many possible complications, their evaluation should be part of an anastomotic healing assessment.

A positive clinical effect could be more pronounced in different experimental settings. Fibrin glue, for example, has been tested in an animal model of complicated colonic anastomosis (severe blood loss, peritonitis), where it showed a positive effect in terms of decreasing morbidity and mortality in the period of 10 postoperative days^52^. The histological assessment of the scar tissue however showed no significant differences between the Control group and the Experimental group on 10th postoperative day measuring also volume fractions of collagen fibers, vWF positive cells and inflammatory reaction^52^ (similarly to our results).

A baseline biopsy has not been taken as no intestine was resected. It can be considered a certain limitation of this study. No clinical nor laboratory signs of any pathologies were, however, evident in our animals and no signs of pathologies were found in the final histological specimens either.

Biocompatibility of the materials used in our study was demonstrated by the presence of the granulation tissue of normal quality (according to all measured qualities) surrounding and invading the material.

The level of biodegradation was not to be measured quantitatively as the degradation process of these nanomaterials was described in previous work^41^.

All laboratory findings were within the physiological range, which suggests that the material does not cause any systemic disturbances. This was expected because the polymers used for the fabrication of the materials have been in use in clinical medicine for many years.

For more discriminating results a study on a complicated anastomosis should be performed as shown in the works of Tebala *et al*.^53^, Zilling *et al*.^44^ and Adas *et al*.^17^.

## Conclusion

We successfully demonstrated that the use of the PCL and PLCL nanofibrous scaffolds in an attempt to fortify an anastomosis on the GI tract is safe. The scaffolds did not influence the amount and quality of scar tissue in the site of anastomosis, and at the same time they did not cause any other kind of complication during our study. From the macroscopic finding they seem to slightly raise the level of adhesions in the site of application, which corresponds to the statement that they promote healing. To be able to assess the adhesion level, we developed and used a novel scoring technique. The material appears to us as a practical and versatile Supporting Material potential of which can be further enhanced for example by adding substances supporting healing like growth factors, antibiotics etc.

The large variability of settings of the fabrication process allows further changes of the material properties.

For the potentially pro-healing qualities of the material, either the PCL or PLCL will be further studied by our team and used as an inner layer of a new double layered patch with an antiadhesive external layer.

## Supplementary information

Dataset 1.

SI Guide.

## Data Availability

The authors declare they will supply any existing additional data as soon as possible when requested.
