# Supplementary material for: Experimental fortification of intestinal anastomoses with nanofibrous materials in a large animal model
Source: Sci Rep. 2020 Jan 24;10:1134. doi: 10.1038/s41598-020-58113-4 (PMC6981151; doi:10.1038/s41598-020-58113-4)
Supplement: Supplementary file 1 — Dataset 1. [file 41598_2020_58113_MOESM1_ESM.pdf]

Experimental fortification of intestinal anastomoses with nano-fibrous materials in a large animal model

Jachym Rosendorf[1,2,], Jana Horakova[4], Marketa Klicova[4], Richard Palek[1,2], Lenka Cervenkova[2], Tomas Kural[3,5], Petr Hosek[2], Tomas Kriz[2], Vaclav Tegl[2,6], Vladimira Moulisova[2], Zbynek Tonar [2,5], Vladislav Treska[1], David Lukas[4], Vaclav Liska[1,2]

1. Department of Surgery, Faculty of Medicine in Pilsen, Charles University, Czech Republic
2. Biomedical Center, Faculty of Medicine in Pilsen, Charles University, Czech Republic
3. Department of Surgery, University Hospital Regensburg, Germany
4. Department of Nonwovens, Faculty of Textile Engineering, Technical University in Liberec
5. Department of Histology and Embryology, Faculty of Medicine in Pilsen, Charles University
6. Department of Anesthesiology and Intensive Care Medicine, Faculty of Medicine in Plzen, Czech Republic

Supplementary table 1

Animal	A1 Zühlke grade	A2 Zühlke grade	A3 Zühlke grade
Control 1	2	2	2
Control 2	0	2	2
Control 3	2	2	2
Control 4	2	2	2
Control 5	2	2	2
Control 6	2	0	0
Control 7	2	2	2
Control 8	0	2	0
PCL 1	2	2	2
PCL 2	2	2	2
PCL 3	2	2	2
PCL 4	2	2	2
PCL 5	2	2	2
PCL 6	2	2	2
PCL 7	0	0	0
PCL 8	0	0	2
PLCL 1	2	2	2
PLCL 2	2	2	2
PLCL 3	2	0	0
PLCL 4	2	2	2
PLCL 5	2	2	0
PLCL 6	2	0	2
PLCL 7	2	2	2
PLCL 8	2	2	2

Table 1: Results of the assessment of quality of the peritoneal adhesions for each anastomosis according to the Zühlke's grading system. Animals are named according to the group allocation. A1 = first anastomosis, A2 = second anastomosis, A3 = third anastomosis.

Supplementary table 2

Animal	A1 PAAS summ	A2 PAAS summ	A3 PAAS summ	PAAS summ for animal
Control 1	3	2	0	5
Control 2	0	3	4	7
Control 3	1	3	0	4
Control 4	4	3	1	8
Control 5	3	4	4	11
Control 6	3	0	0	3
Control 7	3	2	1	6
Control 8	0	2	0	2
PCL 1	5	6	4	15
PCL 2	4	4	6	14
PCL 3	2	1	5	8
PCL 4	5	5	4	14
PCL 5	3	2	3	8
PCL 6	5	4	7	16
PCL 7	0	0	0	0
PCL 8	0	0	4	4
PLCL 1	4	4	3	11
PLCL 2	2	3	3	8
PLCL 3	4	0	0	4
PLCL 4	6	2	2	10
PLCL 5	6	5	0	11
PLCL 6	3	0	4	7
PLCL 7	3	2	3	8
PLCL 8	2	3	2	7

Table 2: Results of the *Perianastomotic adhesions amount scoring* system (PAAS). The table summarizes the total PAAS for each anastomosis and for each animal. Animals are named according to the group allocation. A1 = first anastomosis, A2 = second anastomosis, A3 = third anastomosis.

Supplementary methods 1

Standard operating procedure for peritoneal adhesions quality and quantity assessment, sample collection

---

Created by: *Rosendorf J., Biomedical Center, Faculty of Medicine in Pilsen, Charles University, Czech Republic; verified by Liska V. (Head of the laboratory)*
Date of creation: 10/2017, translated to English 10/2019
Purpose: *Created for the project 'Experimental fortification of intestinal anastomoses with nano-fibrous materials in a large animal model'. Project code: MSMT-26570/2017-2. The aim of this protocol is standardization of sample collection and documentation, peritoneal adhesions qualitative and quantitative assessment (Zühlke grading, Perianastomotic adhesions amount score). The experimental animals are subjected to laparotomy in full anesthesia prior to this procedure.*

Steps:

1. Take a picture of each anastomosis in situ as well as of the whole abdominal cavity to be archived.
2. Release each anastomosis from peritoneal adhesions if present; leave as much adhesion tissue as possible on the intestine while doing so.
3. Evaluate the quality of adhesions for each anastomosis while performing the step number 2 according to Zühlke's grading system for peritoneal adhesions:
    - Grade 0: no adhesions
    - Grade 1: filmy adhesions easy to separate by blunt dissection
    - Grade 2: blunt dissection is possible for the most of adhesions but some sharp dissection is needed, beginning vascularization
    - Grade 3: adhesiolysis is possible by sharp dissection, clear vascularization and bleeding from adhesions during sharp dissection
    - Grade 4: adhesiolysis is possible by sharp dissection but is very challenging. Organs are strongly attached and damage is hardly preventable.
4. Resect each anastomosis together with 2 cm of oral and aboral intestine.
5. Cut the collected sample of intestine immediately after collection longitudinally along the mesentery. Treat the specimens carefully.
6. Pin the sample to a cork underlay with a V-shaped incision in one of its sides (Fig. 1). Mucosa faces the cork. Oral part faces a marked side of the cork underlay.
7. Assign a random alphanumeric code to the anastomosis sample.
8. Take a picture of the anastomosis on the cork underlay with both animal code/number of anastomosis (1/2/3 starting orally) and the new alphanumeric code to be archived.
9. Measure the specimen and divide its circumference into four parts along the suture line. Assign to each quadrant (starting from left to right to respect the polarity of the intestine) a number of points according to the extent of adhesions (Fig. 1):
    - Zero points for no adhesions in the segment
    - One point for a segment being partially covered by adhesions
    - Two points for a fully adhered segment
10. Put the samples of the intestine into 300 ml storing containers filled with 10 % buffered formalin. Mark the containers with the newly created alphanumeric code (for blinding during the histologic evaluation).

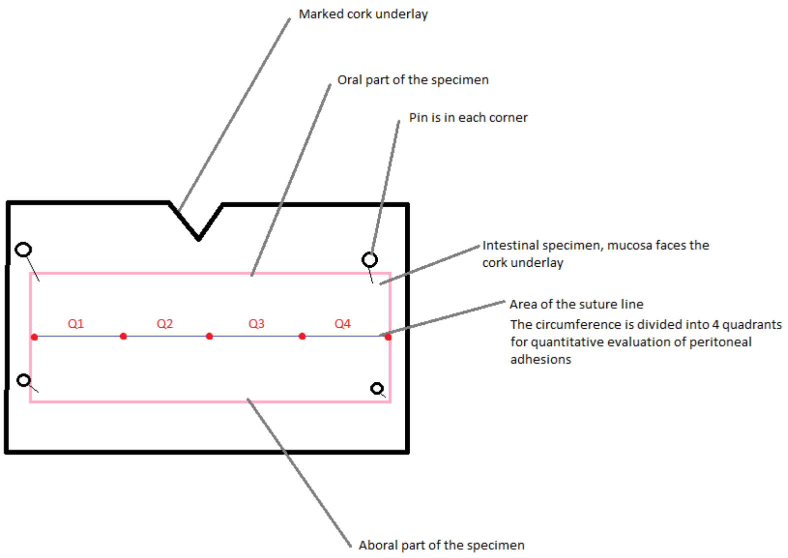

Fig. 1: Scheme of the specimen of the intestine pinned to a cork underlay, divided into four quadrants (Q1-Q4)
