# Supplementary material for: Experimental fortification of intestinal anastomoses with nanofibrous materials in a large animal model
Source: Sci Rep. 2020 Jan 24;10:1134. doi: 10.1038/s41598-020-58113-4 (PMC6981151; doi:10.1038/s41598-020-58113-4)
Supplement: Supplementary file 2 — SI Guide. [file 41598_2020_58113_MOESM2_ESM.docx]

**Experimental fortification of intestinal anastomoses with nano-fibrous materials in a large animal model**

Jachym Rosendorf(1,2,), Jana Horakova(4), Marketa Klicova(4), Richard Palek(1,2), Lenka Cervenkova(2), Tomas Kural(3,5), Petr Hosek(2), Tomas Kriz(2), Vaclav Tegl(2,6), Vladimira Moulisova(2), Zbynek Tonar (2,5), Vladislav Treska(1), David Lukas(4), Vaclav Liska(1,2)

1. Department of Surgery, Faculty of Medicine in Pilsen, Charles University, Czech Republic

2. Biomedical Center, Faculty of Medicine in Pilsen, Charles University, Czech Republic

3. Department of Surgery, University Hospital Regensburg, Germany

4. Department of Nonwovens, Faculty of Textile Engineering, Technical University in Liberec

5. Department of Histology and Embryology, Faculty of Medicine in Pilsen, Charles University

6. Department of Anesthesiology and Intensive Care Medicine, Faculty of Medicine in Plzen, Czech Republic

**List of supplementary files:**

1. **Supplementary information**
   1. **Table 1: Results of the assessment of quality of the peritoneal adhesions for each anastomosis according to the Zühlke’s grading system. Animals are named according to the group allocation. A1 = first anastomosis, A2 = second anastomosis, A3 = third anastomosis.**
   2. **Table 2: Results of the Perianastomotic adhesions amount scoring system. The table summarizes the total PAAS for each anastomosis and for each animal. Animals are named according to the group allocation. A1 = first anastomosis, A2 = second anastomosis, A3 = third anastomosis.**
   3. **Method 1: Standard operating procedure (SOP). This text contains a step-by-step instructions for sample collection and documentation, peritoneal adhesions qualitative and quantitative assessment (Zühlke grading, Perianastomotic adhesions amount score). A schematic figure is included for easier orientation. This SOP was developed and used for this experiment.**
